# Home at Last II: *Gerbera hieracioides* (Kunth) Zardini (Mutisieae, Asteraceae) is really a *Chaptalia*

**DOI:** 10.3897/phytokeys.95.22916

**Published:** 2018-02-09

**Authors:** Xiaodan Xu, Wei Zheng, Vicki A. Funk, Jun Wen

**Affiliations:** 1 Faculty of Art and Communication, Kunming University of Science and Technology, Kunming 650500 China; 2 Faculty of Architecture and City Planning, Kunming University of Science and Technology, Kunming 650500 China; 3 Smithsonian Institution, Department of Botany, MRC 166, Washington, D.C. 20013-7012 USA

**Keywords:** Compositae, *Gerbera hieracioides*, *Trichocline hieracioides*, *Chaptalia hieracioides*, *Gerbera*-complex, SEM, stomata, South America, Africa, Asia

## Abstract

*Gerbera hieracioides* (Kunth) Zardini of the *Gerbera*-complex (Mutisieae, Asteraceae/Compositae) is distributed in Ecuador and Peru. This perennial herb was first named as *Onoseris hieracioides* Kunth and was later recognised as *Trichocline hieracioides* (Kunth) Ferreyra. Now it is generally treated as *Gerbera hieracioides* (Kunth) Zardini but it has never been included in any section of *Gerbera*. In this study, the position of *Gerbera hieracioides* is assessed based on morphology and a molecular phylogeny that includes *G. hieracioides* and 28 other species from the *Gerbera*-complex. Morphologically, *G. hieracioides* bears leaves with the adaxial epidermal surface without stomates but with soft thin trichomes, bracteate scapes, trimorphic capitula and inner ray florets with the corolla shorter than the style. These characters suggest that the species is most closely related to *Chaptalia* rather than to *Gerbera* or *Trichocline*. Furthermore, the phylogenetic results based on two nuclear (ITS and ETS) and two chloroplast (*trn*L–*trn*F and *trn*L–*rpl*32) sequences strongly support the placement of *G. hieracioides* nested within *Chaptalia*. As both morphological characters and the molecular phylogenetic results support the transfer of *G. hieracioides* to *Chaptalia*, this enigmatic taxon is recognised as *Chaptalia hieracioides* (Kunth) X.-D. Xu & W. Zheng.

## Introduction

*Gerbera hieracioides* (Kunth) Zardini (Mutisieae, Asteraceae) is a species belonging to the *Gerbera*-complex (*Gerbera* L., *Leibnitzia* Cass., *Uechtritzia* Freyn, *Amblysperma* Benth., *Chaptalia* Vent., *Trichocline* Cass., *Perdicium* L. and *Lulia* Zardini). The species is distributed in Ecuador and Peru. This perennial herb was first named as *Onoseris hieracioides* Kunth in 1818. It was transferred to *Trichocline hieracioides* (Kunth) Ferreyra in 1944. In 1974, Zardini moved this species out of *Trichocline* because it did not have the characters which were used to define that genus. The apex of achenes is truncate in *Trichocline* but tapering or beaked in *G. hieracioides* (Zardini 1974, Hansen 1990). Zardini (1974, 1975) moved it into *Gerbera* because it had bracteate scapes, uniseriate ray florets, achenes rostrate at the apex and slender achene hairs. However, *Gerbera* and *Chaptalia* were found to share the same traits such as achenes rostrate at the apex (Katinas 2004) and the transfer of *Trichocline hieracioides* to *Gerbera* remained controversial (Hansen 2006).

*Gerbera* currently contains about 32 species, which belong to six sections: the three African sections: *Gerbera* (8 species), *Parva* H.V.Hansen (1 species) and *Lasiopus* (Cass.) Sch.Bip. (6 species), the Aisan section Isanthus (Less.) C. Jeffrey (7 species), the Madagascar section Pseudoseris (Baill.) C. Jeffry (8 species) and section Piloselloides Less. (2 species, one of which is widespread from Asia, Africa and Australia: Hansen 1985a, 1985b, 1988, Johnson et al. 2014, Funk et al. 2016). However, Zardini (1974) did not include *G. hieracioides* in any section of *Gerbera*, she only compared it with two species in sect. Lasiopus (Hansen 1988): *G. jamesonii* Bolus ex Adlam and *G. ambigua* Sch. Bip. Although *G. hieracioides* has the trimorphic capitula similar to those of G. sect. Lasiopus (Hansen 1985a) from Africa, it has bracteate scapes, suggesting that it is perhaps related to Gerbera sect. Isanthus (Hansen 1988) from Asia. Furthermore, the SEM studies showed that the achene hairs of *G. hieracioides* possess a significantly lower L/W ratio than that in either sect. Isanthus or sect. Lasiopus of *Gerbera* (Hansen 1990). Therefore, it was still difficult to place *G. hieracioides* into an existing section (or a new section) of the genus *Gerbera*.

*Gerbera* is an Old World genus, whereas *Chaptalia*, *Trichocline* and the enigmatic *G. hieracioides* are New World groups (Nesom 2004b, 1995). Recently, phylogenetic analyses of the *Gerbera*-complex based on molecular data showed that *Chaptalia* was placed between *Trichocline* and *Gerbera* (Baird et al. 2010, Funk et al. 2014, Pasini et al. 2016). This suggested to the authors that the New World *G. hieracioides* may be a species of *Chaptalia*.

In this study, the authors seek to determine the correct generic placement of *G. hieracioides* by sampling 28 congeneric species using both molecular (two nuclear and two chloroplast markers) and morphological data (leaf adaxial surface, scape and floral morphology).

## Materials and methods

A total of 29 species from four genera (*Gerbera*, *Amblysperma*, *Chaptalia* and *Trichocline*) of the *Gerbera* complex and *Adenocaulon chilense* (outgroup) were sampled for this study. Most of the specimens were sampled from the United States National Herbarium (US) of the Smithsonian Institution (Tables 1, 2).

**Table 1.** Voucher information and morphological characters of *Gerbera hieracioides* and the related species.

Species	Section	Locality	Voucher information	Adaxial leaf		Bracts on scape	Inner rays
				Stomata	Trichome		
*Gerbera viridifolia* (DC.) Sch.Bip.	*Lasiopus*	Kenya	*T.H. Trinder-Smith s.n.* (US)	+	★	−	+
*G. jamesonii* Adlam	*Lasiopus*	Cultivar	*V.A. Funk s.n.* (US)	+	★	−	+
*G. aurantiaca* Sch.Bip.	*Lasiopus*	South Africa	*Bayliss 2505* (US)	+	★	−	+
*G. ambigua* Sch.Bip.	*Lasiopus*	South Africa	*M. Koekemoer 2097* (US)	+	★	−	+
*G. piloselloides* Cass.	*Piloselloides*	Swaziland	*M. Koekemoer 2590* (US)	+	★	−	+
*G. cordata* Less.	*Piloselloides*	Madagascar	*T.B. Croat 29083* (MO)	+	★	−	+
*G. perrieri* Humbert	*Pseudoseris*	Madagascar	*L. Gautier 3110* (MO)	+	★	−	+
*G. crocea* Kuntze	*Gerbera*	South Africa	*M. Koekemoer 2029* (US)	+	≈	+	−
*G. wrightii* Harv.	*Gerbera*	South Africa	*P. Goldblatt 5287* (US)	+	≈	+	−
*G. serrata* Druce	*Gerbera*	South Africa	*M. Koekemoer 2001* (PRE)	+	≈	+	−
*G. gossypina* Beauverd	*Isanthus*	India	*W.N. Koelz 4828* (US)	−	−	+	−
*G. maxima* Beauverd	*Isanthus*	India	*D.H. Nicolson 2755* (US)	−	−	+	−
*G. delavayi* Franch.	*Isanthus*	China	*X. Xu 1102* (KMUST)	−	−	+	−
*G. nivea* Sch.Bip.	*Isanthus*	China	*J.F. Rock 6430* (US)	−	−	+	−
*G. henryi* Dunn	*Isanthus*	China	*W.B. Hemsley 1903* (US)	−	−	+	−
*G. hieracioides* (Kunth) Zardini	?	Ecuador	*P.M. Peterson 9287* (US)	−	≈	+	+
*G. hieracioides* (Kunth) Zardini	?	Peru	*R. Ferreyra 15362* (US)	−	≈	+	+
*Chaptalia pringlei* Greene	N	Mexico	*Rzedowski 34853* (US)	−	≈	+	+
*C. mandonii* Burkart	N	Argentina	*P.M. Simón 438* (US)	−	≈	+	+
*C. meridensis* S.F. Blake	N	Venezuela	*L. Aristeguieta 2591* (US)	−	≈	+	+
*Trichocline cineraria* Hook. & Arn.	N	Argentina	*A.R. Cuezzo 20mz398* (US)	+	≈	+	-
*T. catharinensis* Cabrera	N	Brazil	*L.B. Smith 11376* (US)	+	≈	+	-

Notes: + designates those mentioned present; − designates those mentioned absent; ★ designates rigid, straight and upright trichomes present on the adaxial leaf surface; ≈ designates soft thin trichomes present on the adaxial leaf surface; N designates data not available.

**Table 2.** Voucher information and GenBank accessions of *Gerbera hieracioides* and the related species.

Species	Locality	Voucher information	ITS	ETS	*trn*L–*trn*F	*trn*L–*rpl*32
*Gerbera viridifolia* (DC.) Sch. Bip.	South Africa	*T.H. Trinder-Smith s.n.* (US)	MG661696*	MG661588*	MG661639*	MG661670*
*G. crocea* Kuntze	South Africa	*M. Koekemoer 2029* (US)	MG661709*	MG661606*	MG661645*	MG661683*
*G. delavayi* Franch.	China	*X. Xu 1102* (KMUST)	MG661708*	MG661605*	MG661659*	MG661682*
*G. henryi* Dunn	China	*X. Xu 1103* (KMUST)	MG661706*	MG661602*	MG661655*	MG661681*
*G. nivea* Sch. Bip.	China	*Y.S. Chen 2674* (PE)	MG661703*	MG661598*	MG661648*	MG661678*
*G. aurantiaca* Sch.Bip.	South Africa	*Bayliss 2505* (US)	MG661711*	MG661610*	MG661637*	MG661687*
*G. ambigua* Sch. Bip.	South Africa	*M. Koekemoer 2097* (US)	MG661712*	MG661611*	MG661636*	MG661688*
*G. jamesonii* Adlam	Cultivar	*T. Derby s.n.* (US)	MG661704*	MG661599*	MG661638*	MG661679*
*G. cordata* Less.	South Africa	*J. Wen 10067* (US)	N	MG661608*	MG661661*	MG661685*
*G. piloselloides* Cass.	Swaziland	*M. Koekemoer 2590* (US)	MG661701*	MG661592*	MG661650*	MG661675*
*G. wrightii* Harv.	South Africa	*P. Goldblatt 5287* (US)	MG661695*	MG661587*	MG661642*	N
*G. serrata* Druce	South Africa	*M. Koekemoer 2001* (PRE)	MG661697*	MG661590*	MG661656*	MG661671*
*G. hieracioides* (Kunth) Zardini	Ecuador	*P.M. Peterson 9287* (US)	MG661705*	MG661601*	MG661657*	MG661680*
*G. hieracioides* (Kunth) Zardini	Peru	*J. Campos 5255* (US)	N	MG661600*	N	N
*Amblysperma scapigera* Benth.	Australia	*A. Morrison s.n.* (US)	MG661713*	MG661612*	N	MG661689*
*Adenocaulon chilense* Less.	Chile	*G.L. Sobel 2558* (US)	MG661714*	N	N	MG661690*
*Gerbera maxima* Beauverd	India	*F. Kingdom 18199* (NY)	KX349402	N	KX349371	N
*G. gossypina* Beauverd	India	*W. Koelz 4294* (US)	GU126777	N	N	GU126755
*Adenocaulon chilense* Less.	Argentina	*J.M. Bonifacino 3997* (LP)	KX349359	N	KX349360	N
*Chaptalia nutans* (L.) Polák	Argentina	*P.M. Simon 477* (US)	GU126772	N	N	GU126751
*C. pringlei* Greene	Mexico	*G. Nesom 4405* (US)	GU126773	N	N	N
*C. runcinata* Kuntze	Argentina	*P.M. Simon 415* (US)	GU126774	N	N	GU126752
*C. chapadensis* D.J.N. Hind	Argentina	*Roque & al. 2188* (ALCB)	KF989508	N	KF989614	N
*C. similis* R.E. Fr.	Argentina	*P.M. Simon 711* (US)	GU126775	N	N	GU126753
*C. tomentosa* Vent.	USA	*V.A. Funk 12303* (US)	GU126776	N	N	GU126754
*C. piloselloides* (Vahl) Baker	Brazil	*E. Pasini 1021* (ICN)	KX349357	N	KX349358	KX349403
*Trichocline auriculata* Hieron	Argentina	*H. Simón & J.M. Bonifacino 633* (US)	KX349386	N	KX349387	N
*T. catharinensis* Cabrera	Brazil	*E. Pasini 915* (ICN)	KX349388	N	KX349389	KX349411
*T. caulescens* Phil.	Chile	*V.A. Funk & al. 13055* (US)	KX349390	N	KX349391	KX349406
*T. cineraria* Hook. & Arn.	Argentina	*E. Pasini & F. Torchelsen 1027* (ICN)	KX349392	N	KX349393	KX349407
*T. plicata* Hook. & Arn.	Argentina	*E. Pasini & F. Torchelsen 1023* (ICN)	KX349396	N	KX349397	KX349409
*T. reptans* (Wedd.) Hieron	Argentina	*E. Pasini & F. Torchelsen 1025* (ICN)	KX349398	N	KX349399	KX349410

Notes: * designates the new sequences from this study; N designates data not available.

**Adaxial leaf epidermal morphology.** Lamina (0.5–1.0 cm^2^) were placed with the adaxial side exposed on carbon tape over stubs for the scanning electron microscopy (SEM), without soaking the material in different solutions prior to SEM. The stubs bearing leaves were treated with gold-palladium to 16.6 μm thickness and were examined under a Philips XL-30 scanning electron microscope at the SEM Lab of the National Museum of Natural History (NMNH). The 22 samples were subsequently observed and photographed under SEM. Images of the leaves were captured using the proprietary software associated with the Philips SEM. Images of at least 15 different areas of the adaxial leaf surface were captured.

**Floret morphology.** The florets and scapes of 20 herbarium specimens were examined in the United States National Herbarium, Smithsonian Institution, using an optical microscope.

**DNA extraction, amplification and sequencing.** The molecular work was performed in the Laboratory of Analytical Biology (LAB) of NMNH (Smithsonian Institution). DNAs of 16 samples (15 species, including two samples of *Gerbera hieracioides*) were extracted using the AutoGen. Herbarium leaf samples, along with 1.0 and 2.3 mm diameter beads, were dipped in liquid nitrogen then immediately shaken for 30 seconds at 18000 rpm. About 500 ml of CTAB was added to the tubes, vortexed and incubated overnight (500 rpm at 45 °C). Then 300 µl of the supernatant was transferred to an AutoGen plate. AutoGen was run according to the manufacturer’s default settings (AutoGen, Inc., Holliston, MA, USA).

Four markers including two nuclear ribosomal (ITS and ETS) and two chloroplast intergenic spacers (*trn*L–*trn*F and *trn*L–*rpl*32) were amplified. The ITS primers were designed by Downie and Katz-Downie (1996) and White et al. (1990), ETS primers by Baldwin and Markos (1998), *trn*L–*trn*F primers by Taberlet et al. (1991) and *trn*L–*rpl*32 spacer primers by Timme et al. (2007) (Table 3). The PCR reaction mixture had a total volume of 25 µl, comprising 14.05 µl nuclease free water, 2.5 µl 10× buffer, 2 µl dNTPs, 1.25 µl MgCl_2_, 1 µl of both forward and reverse primers, 0.5 µl BSA, 0.2 µl Taq DNA polymerase and 2.5 µl of template DNA. The amplified products were purified with ExoSapIT enzyme with activation at 37 °C and deactivation at 95 °C. 4 µl of the purified product and same primers (1 µl, 1 µM) were cycle-sequenced in a mixture containing 0.8 µl Big Dye (Applied Biosystems, Foster City, USA) and 2.0 µl 5× Big Dye buffer and 4.2 µl water.

**Table 3.** Primers and amplification protocols for all markers.

Marker	Primers and sequences 5′–3′	PCR protocol: initial pre-heating; DNA denaturation; primer annealing; DNA extension; final extension
ITS	ITS5A: GGAAGGAGAAGTCGTAACAAGGITS4: TCCTCCGCTTATTGATATGC	95 °C 1 min; 54 °C 1 min; 72 °C 1 min; 72 °C 10 min; 40 cycles
ETS	18s-ETS: ACTTACACATGCATGGCTTAATCTETS-Hel-1: GCTCTTTGCTTGCGCAACAACT	94 °C 0:30 min; 60 °C 0:40 min; 72 °C 1:20 min; 72 °C 5 min; 30 cycles
*trn*L–*trn*F	*trn*L-Fc: CGAAATCGGTAGACGCTACG*trn*L-Ff: ATTTGAACTGGTGACACGAG	94 °C 1 min; 53 °C 1 min; 72 °C 2 min; 72 °C 10 min; 35 cycles
*trn*L–*rpl*32	*trn*L: TACCGATTTCACCATAGCGG*rpl*32: AGGAAAGGATATTGGGCGG	95 °C 3 min; 51 °C 40 s; 72 °C 1:20 min; 72 °C 5 min; 40 cycles

The cycle sequencing programme was 30 cycles of 95 °C for 30 s, 50 °C for 30 s and 60 °C for 4 min. The resultant product was sephadex filtered and sequenced through an ABI 3730 automated sequencer (Applied Biosystems, Foster City, USA). The PCR reactions were performed in a Veriti PCR Thermal Cycler. The amplification protocols for all markers are summarised in Table 3. Sequences were aligned by using MAFFT (Katoh and Standley 2013) using Geneious 10.0.9. (Biomatters Ltd., Auckland, New Zealand) and checked manually. A total of 57 newly generated sequences from the 16 samples were deposited in GenBank (Table 2).

A total of 37 sequences of 16 species were retrieved from NCBI for the related taxa within the tribe Mutisieae (Table 2). Phylogenetic relationships were inferred based on the concatenated ITS+ETS+*trn*L–*rpl*32+*trn*L–*trn*F data with MrBayes v. 3.2.2 (Ronquist et al. 2012) by using the substitution model of GTR based on the best-fitting model determined using jModelTest 2.1.6 (Posada 2008), the chain length of 10,000,000, rate variation of gamma, gamma categories of 4, heated chains of 4, heated chain temp. of 0.2, subsampling freq. of 200 and burn-in length of 100,000. Tracer v. 1.5 (Rambaut and Drummond 2009) was used to confirm that the effective sample size (ESS) for all relevant parameters was > 200. After discarding the trees as burn-in, a 50 % majority-rule consensus tree and posterior probabilities (PP) for node support were calculated using the remaining trees.

## Results

**Figure 1.**
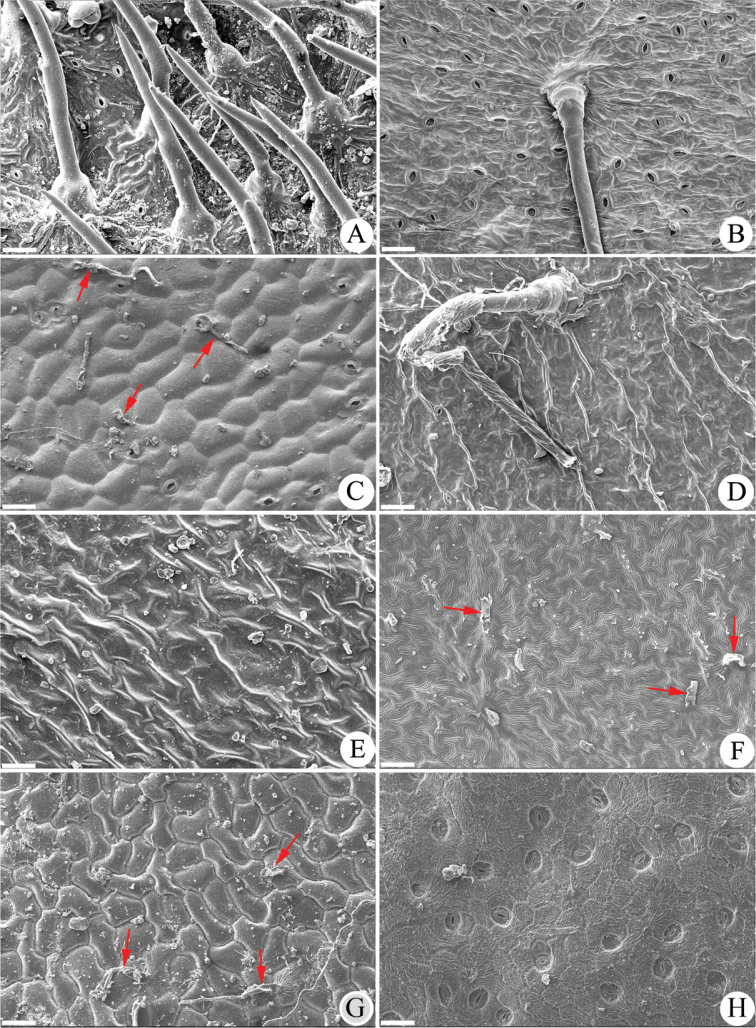
Adaxial leaf epidermal surface morphology of *Gerbera hieracioides* and the related species. **A** *G. ambigua* (sect. Lasiopus) **B** *G. piloselloides* (sect. Piloselloides) **C** *G. crocea* (sect. Gerbera) **D** *G. perrieri* (sect. Pseudoseris) **E** *G. maxima* (sect. Isanthus) **F** *Chaptalia pringlei* **G** *G. hieracioides* **H** *Trichocline catharinensis*. Arrows point to the soft thin trichomes. Scale bar=50 μm.

**Adaxial leaf epidermal morphology.** The results of the SEM work (Table 1) showed that the two tested samples of *Gerbera hieracioides* have no stomates but have soft, thin and appressed trichomes on the adaxial leaf surface (Figure 1G). These adaxial leaf morphological traits differ from the *Gerbera* species: (1) they are different from *Gerbera* sections sampled [sect. Lasiopus (4 species), sect. Piloselloides (2 species) and sect. Pseudoseris (1 species)] which have stomates and stiff, straight, upright trichomes. Figure 1 has images of one sample for each section: *G. ambigua* (Fig. 1A), *G. piloselloides* (Fig. 1B) and *G. perrieri* (Fig. 1D), respectively. (2) they are different from the members of Gerbera sect. Gerbera which have stomates and soft, thin and appressed trichomes. Three species from South Africa were examined and represented by *G. crocea* (Fig. 1C). (3) they are different from the Asian Gerbera sect. Isanthus which have no stomates and no trichomes based on this study of five species of sect. Isanthus that were examined in the study and are represented by *G. maxima* (Fig. 1E): the authors’ observations agree with Lin et al. (2008) for the Asian species *G. delavayi*. Additionally, the morphological traits of *G. hieracioides* differ significantly from those of the *Trichocline* species, which have many stomates with guard cells sunken on the leaf surface, illustrated by *T. catharinensis* (Fig. 1H). However, the two tested *G. hieracioides* samples share the same adaxial leaf epidermal characters such as soft, thin and appressed trichomes, epidermal cell shape and striations and absence of stomates, with the three examined *Chaptalia* species, as represented by *C. pringlei* (Fig. 1F). Therefore, based on the adaxial leaf epidermal morphology, *G. hieracioides* is most closely related to *Chaptalia* rather than to *Gerbera* or *Trichocline*.

**Figure 2.**
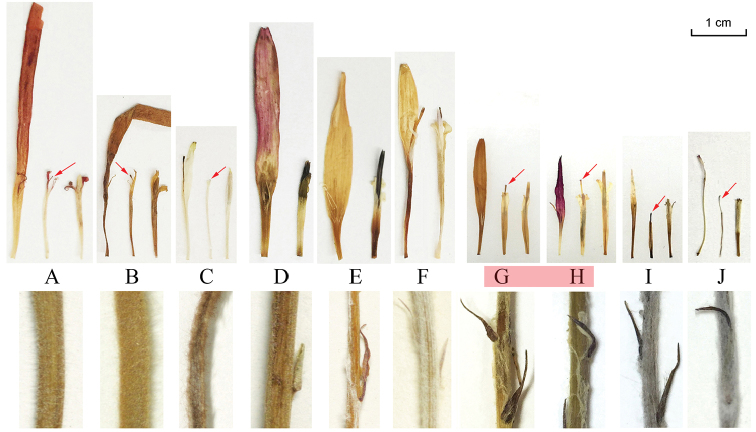
Scape and floret morphology of *Gerbera hieracioides* and the related species. **A** *G. jamesonii* (sect. Lasiopus) **B** *G. ambigua* (sect. Lasiopus) **C** *G. cordata* (sect. Piloselloides) **D** *G. crocea* (sect. Gerbera) **E** *G. nivea* (sect. Isanthus) **F** *G. gossypina* (sect. Isanthus) **G** *G. hieracioides* (Ecuador) **H** *G. hieracioides* (Peru) **I** *Chaptalia meridensis* **J** *C. mandonii*. The arrows mark the styles of inner ray florets.

**Scape and floret morphology.** The results (Table 1) showed that the two examined samples of *Gerbera hieracioides* have bracteate scapes and trimorphic capitula which have the inner rays with corollae shorter than the styles (Fig. 2G, H). The above morphological traits also differ from those of the *Gerbera* species: (1) Gerbera sect. Lasiopus, sect. Piloselloides and sect. Pseudoseris have ebracteate scapes and trimorphic capitula and the inner rays have corollae as long as the styles or longer. *Gerbera jamesonii* (Fig. 2A) and *G. ambigua* (Fig. 2B) belong to sect. Lasiopus and *G. cordata* (Fig. 2C) for sect. Piloselloides. (2) they are different from Gerbera sect. Gerbera and sect. Isanthus, which have bracteate scapes but dimorphic capitula without inner rays of florets. Three South African species and five Asian species were examined and are illustrated by *G. crocea* (Fig. 2D), *G. nivea* (Fig. 2E) and *G. gossypina* (Fig. 2F). The two tested *G. hieracioides* samples share the traits of bracteate scapes and trimorphic capitula which have inner rays with corollae shorter than the styles with the three tested *Chaptalia* species, represented by *C. meridensis* (Fig. 2I) and *C. mandonii* (Fig. 2J). Therefore, based on the scape and floret morphology, *G. hieracioides* should be best considered as a species of *Chaptalia* rather than *Gerbera*.

**Figure 3.**
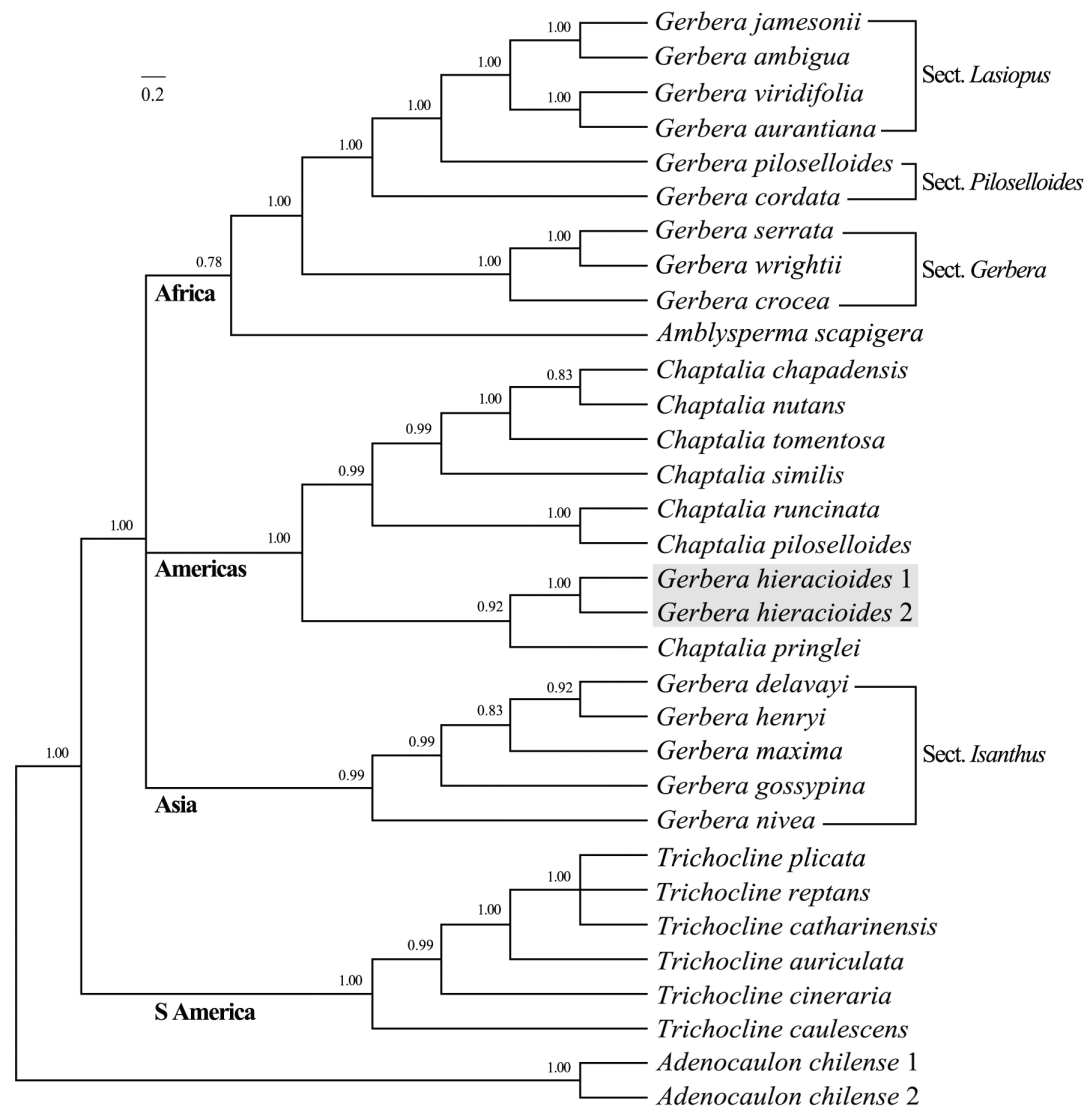
Phylogeny of *Gerbera hieracioides* and the related species. The phylogeny is based on the MrBayes analysis of the combined ITS and ETS, *trn*L–*trn*F and *trn*L–*rpl*32 markers. The posterior probabilities support values are shown next to branches.

**Phylogenetic analysis.** The MrBayes analysis of the combined nuclear markers and two plastid genes showed four clades of the sampled species of the *Gerbera*-complex, all with a strong biogeographic signal (Fig. 3): (1) the African and Australian species of the *Gerbera* complex (African *Gerbera* species are sister to the Australian *Amblysperma*), (2) the American genus *Chaptalia* and the South American *Gerbera hieracioides*, (3) the Asian *Gerbera* species and (4) the South American genus *Trichocline*. However, there is no well-supported resolution amongst the first three clades mentioned above, so no conclusions can be made about the monophyly of *Gerbera* at this time.

Both samples of *Gerbera hieracioides* were nested within the *Chaptalia* clade. *Gerbera hieracioides* is sister to *Chaptalia pringlei*; then the *G. hieracioides-C. pringlei* clade is sister to the other *Chaptalia* species with strong support (posterior probability of 1.00). Therefore, the molecular data also support the placement of *G. hieracioides* in *Chaptalia*.

### Discussion

The molecular phylogeny of the *Gerbera*-complex showed that *G. hieracioides* did not group with *Trichocline* (Fig. 3) but was nested inside *Chaptalia*. Furthermore, the leaf adaxial epidermis of *G. hieracioides* has no stomates, while that of *Trichocline* usually has many stomates (Fig. 1). In addition, Katinas (2004) presented a key to distinguish the genera of the *Gerbera*-complex and *Gerbera* and *Chaptalia* were found to share the same trait of achenes rostrate at the apex but this is not found in *Trichocline*.

The confusion about the placement of *Gerbera hieracioides* is no doubt the result of the morphology falling between that of *Gerbera* and *Chaptalia*. A good case concerning this point is the inner ray florets of the trimorphic capitula: *Gerbera* has a corolla as long as the style or longer and the staminodes are present, whereas *Chaptalia* has the corolla shorter than the style and without staminodes (Katinas 2004). As for *G. hieracioides*, the inner ray florets have moderately reduced stamens (Fig. 2G, H) which are different from both *Gerbera* and *Chaptalia* species. Although the stamen morphology of *G. hieracioides* is not identical to *Chaptalia*, their moderately reduced corollae (Fig. 2G, H) are similar to those of *Chaptalia* rather than those of *Gerbera*, according to Katinas (2004). Furthermore, the characters of leaf adaxial epidermis of *G. hieracioides* including the lack of stomates and the presence of soft thin trichomes, as well as bracteate scapes and cell shape and striations, all suggest that the species is closest to *Chaptalia*. Additionally, Hansen (1990) stated that the achene hairs of *G. hieracioides* are sub-inflated with a lower L/W-ratio than that of *Gerbera*. Therefore, the morphological data support the transfer of *G. hieracioides* to *Chaptalia* that was consistent with the molecular phylogeny (Fig. 3) based on both nuclear ITS and ETS and chloroplast *trn*L–*trn*F and *trn*L–*rpl*32. This transfer is in agreement with the geographic distribution (Fig. 3), because *G. hieracioides* is from South America and all the other *Chaptalia* species are from the New World (Nesom 2004b, 1995).

*Chaptalia* is a New World genus and contains about 70 species in the Americas (Funk et al. 2016). Although there are partial regional treatments, there is no comprehensive monograph of the genus (e.g. Burkart 1944, Cabrera and Nesom 2003, Nesom 2004a, b). Hansen (2006) argued that the most significant problem of the *Gerbera*-complex is the lack of a revisionary treatment of *Chaptalia* and argued for further studies to test whether *Chaptalia* is monophyletic. In the molecular analysis (Fig. 3), the nine *Chaptalia* samples (including *G. hieracioides*) grouped into two well-supported clades. This result indicates that *Chaptalia* seems to be monophyletic when *G. hieracioides* is included. *Chaptalia* is typically characterised by differentiated and reduced rays (Hansen 1990): the inner ray florets with corolla strongly reduced, filiform (irregularly tubular, ligulate or bilabiate), shorter than the style and without staminodes (Katinas 2004). The inner ray florets of *G. hieracioides* with moderately reduced corollae and stamens suggest that the inner ray florets of trimorphic capitula may be a key morphological character for the further revisionary treatment of *Chaptalia*.

As for *Gerbera*, this study showed that it falls into two distinct clades, one from Africa which is the sister group of the Australian genus *Amblysperma* and the other contains all the Asian *Gerbera* (Fig. 3). However, the two *Gerbera* clades are in a trichotomy with the *Chaptalia* clade. It is clear that, based on the sampling, the Asian taxa may be best separated out into a separate genus then *Amblysperma* is the sister genus of African *Gerbera*. If the two clades of *Gerbera* form a single clade, then *Amblysperma* will most likely be nested within that clade. The decision must wait for ongoing studies using additional data. However, it is clear that *Gerbera hieracioides* should be considered within *Chaptalia*.

### Taxonomic treatment

#### Chaptalia hieracioides

Taxon classificationPlantaeAsteralesAsteraceae

(Kunth) X.-D.Xu & W.Zheng comb. nov.

urn:lsid:ipni.org:names:60476046-2

Basionym: Onoseris hieracioides Kunth, Nov. Gen. Sp. [H. B. K.] 4 (ed. folio): 5, Tab. 304. 1818; 4 (ed. quarto): 7, Tab. 304. 1820. **Type**: Ecuador: “Alousi”, A.J.A. Bonpland 3233 (Lectotype: P00322236, here designated).
Trichocline peruviana Hieron., Bot. Jahrb. Syst. 21: 368. 1895. [according to IPNI]
Trichocline hieracioides (Kunth) Ferreyra, J. Arnold Arbor. 25: 394. 1944, comb. illeg. non Baker (1884).
Gerbera hieracioides (Kunth) Zardini, Bol. Soc. Argent. Bot. 16(1–2): 105. 1974.
Trichocline beckerae (as ‘beckeri’) H.Rob., Phytologia 65(1): 47. 1988.

## Conclusions

The placement of *Gerbera hieracioides* within *Chaptalia* is strongly supported by both the molecular sequence data (two nuclear markers ITS and ETS and two chloroplast markers *trn*L–*trn*F and *trn*L–*rpl*32) and the morphology of the scape, capitula and the leaf adaxial epidermal surface. Therefore, *Gerbera hieracioides* has been transferred to *Chaptalia* and it is recognised as *Chaptalia hieracioides* (Kunth) X.-D. Xu et W. Zheng.

## Supplementary Material

XML Treatment for Chaptalia hieracioides
